# Manejo de glomerulonefritis membranoproliferativa secundaria a infección por virus de hepatitis C

**DOI:** 10.23938/ASSN.1055

**Published:** 2023-11-15

**Authors:** Ignacio Lasierra Lavilla, Amalia Perona Caro, Celia del Agua Arias-Camisón, Julien Paola Caballero Castro

**Affiliations:** 1 Servicio Aragonés de Salud Hospital Obispo Polanco Servicio de Medicina Interna Teruel España; 2 Servicio Aragonés de Salud Hospital Obispo Polanco Servicio de Nefrología Teruel España; 3 Servicio Aragonés de Salud Clínico Universitario Lozano Blesa Servicio de Anatomía Patológica Zaragoza España

**Keywords:** Glomerulonefritis membranoproleferativa, Virus de la Hepatitis C, Antivirales, Corticoesteroides, Eritrocitos anormales, Membranoproliferative glomerulonephritis, Hepatitis C virus, Glecaprevir-Pibrentasvir, Corticoids, Abnormal erythrocytes

## Abstract

Las glomerulonefritis membranoproliferativas son nefropatías glomerulares poco frecuentes cuya prevalencia ha disminuido en nuestro medio. Presentan una histología característica y pueden asociarse a diferentes enfermedades. La presentación clínica es variada y su diagnóstico definitivo requiere realizar una biopsia renal. El tratamiento viene condicionado por la enfermedad de base, tratándose con inmunosupresores cuando existe una disminución del filtrado glomerular.

Presentamos el caso de una mujer de 47 años con glomerulonefritis membranoproliferativa secundaria a infección por de virus de hepatitis C para describir el manejo de este tipo de pacientes, dado que se trata de una patología con una baja prevalencia.

## Abstract (en)

Membranoproliferative glomerulonephritis is an uncommon condition that affects the glomeruli of the kidneys; its prevalence has decreased in our environment. Membranoproliferative glomerulonephritis has a characteristic histology that can be associated to different diseases. The clinical presentation varies, and to achieve a definitive diagnosis a renal biopsy must be done. Treatment is based on the underlying disease; when a drop in glomerular filtration rate is detected, immunosuppressants are prescribed.

We describe the management of a 47-year-old female with membranoproliferative glomerulonephritis secondary to hepatitis C virus infection, a condition with very low prevalence.

## INTRODUCCIÓN

La glomerulonefritis membranoproliferativa (GnMP) incluye un grupo de nefropatías glomerulares poco frecuentes con un patrón histológico de lesión glomerular característico^1^. Esta entidad puede verse asociada a: infecciones (siendo la más frecuente la del virus de la hepatitis C, VHC), enfermedades autoinmunes o gammapatías monoclonales^1^. La prevalencia de GnMP ha disminuido hasta un 1-4% en los países desarrollados, probablemente debido a los nuevos tratamientos para la infección por VHC^2^.

La presentación clínica es variada, desde hematuria con o sin proteinuria hasta un síndrome nefrótico^3^. El diagnóstico definitivo requiere realizar una biopsia renal y un estudio de inmunofluorescencia^4^, clasificando la GnMP en GnMP mediada por inmunocomplejos y GnMP mediada por complemento^4^. El tratamiento está condicionado por la enfermedad de base. En casos que cursen con una importante disminución del filtrado glomerular el tratamiento de elección son los corticoesteroides, pudiendo añadir un segundo fármaco inmunosupresor si fuese necesario^3^.

Presentamos el caso de una mujer de 47 años que presentó una GnMP secundaria a una infección por VHC, patología con una baja prevalencia, que presentó una excelente respuesta al tratamiento con corticoides y antivirales.

## CASO CLÍNICO

Mujer de 47 años que ingresó en el servicio de Medicina Interna de nuestro hospital desde Urgencias, donde había acudido por cuadro de malestar general, cefalea occipital, dolor epigástrico y náuseas de una semana de evolución que fueron incrementándose de forma progresiva. La paciente presentó también aumento de perímetro en extremidades inferiores (EEII) y cifras de presión arterial elevadas (en torno a 100-150 mm Hg). En la exploración destacó la presencia de edema en EEII y un exantema cutáneo con lesiones petequiales generalizadas, con predominio en tórax y extremidades inferiores.

Al ingreso se le realizó una analítica sanguínea en la que destacaban valores elevados de propéptido natriurético (pro-BNP) y creatinina (2 mg/dL), elavación de reactantes de fase aguda y proteinuria en rango nefrótico (8,8 g/L). Se observó hematuria y en el urosedimento se detectaron abundantes hematíes dismórficos (40%) (Tabla 1).

**Tabla 1:** Evolución de los parámetros bioquímicos sanguíneos y morfológicos del sedimento urinario

	Analítica		
	Ingreso	Alta	Seguimiento
*Sangre*			
Creatinina (mg/dL)	2	0,7	0,8
Proteínas (mg/L)	4,6	4,3	6,2
Albúmina (mg/L)	2,4	2,5	3,9
Colesterol total (mg/dL)	140	365	197
LDL colesterol (mg/dL)	82,5	262,6	107
Carga viral VHC (UI/mL)	458.000	194.000	Negativa
C3 Complemento (mg/dL)	60	93	100
C4 Complemento (mg/dL)	2	8	16
*Orina*			
Hematíes por cga	>50	out/25	01/mai
Hematíes dismórficos	40%	26%	0%
Proteinuria (g/L)	8,8	4,8	0,16

Ingreso: tras una semana de sintomatología; clínica), Alta: tras tres semanas de ingreso, iniciados tratamientos con corticoides y antiviral; Seguimiento: en consultas externas, tras finalizar tratamiento antiviral frente a virus de la hepatitis C; LDL: lipoproteínas de baja densidad; VHC: virus de la hepatitis C; C: componente; cga: campos de gran aumento (40x).

Se sospechó patología glomerular con predominio de síndrome nefrótico, siendo la glomerulonefritis membranosa y la GnMP las más compatibles. Dado el deterioro de la filtración glomerular, se inició tratamiento con corticoides a dosis intermedias (bolos 120 de mg al día, en pauta descendente), y se realizaron una biopsia renal y otra de tipo *punch* de las lesiones cutáneas de las EEII, que mejoraron tras la administración de los corticoides.

También se realizaron estudios de autoinmunidad y de electroforesis, ambos con resultado normal, y de serología vírica, donde se detectó una carga viral de 458.000 UI/mL (log 5,66) para el VHC. Esta positividad, que no era conocida previamente, motivó iniciar tratamiento para VHC con Glecaprevir/Pibrentasvir 100mg/40mg, tres comprimidos al día durante ocho semanas.

**Figura 1:**
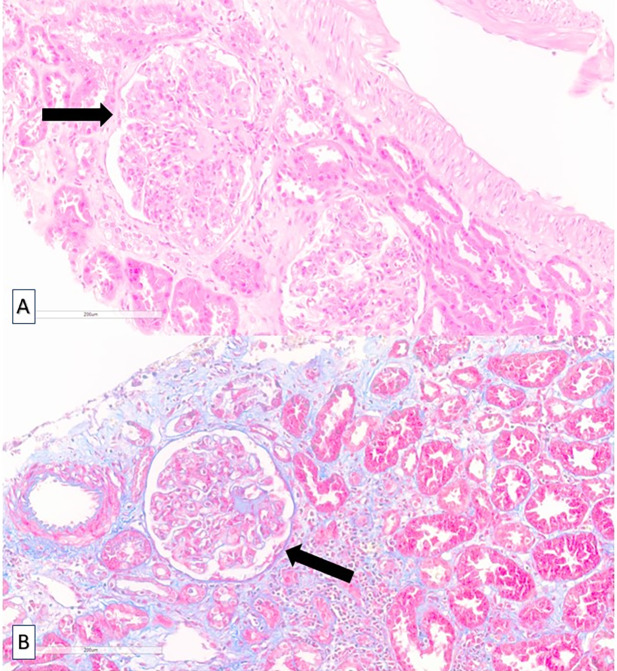
Microscopía óptica. Glomérulo de aspecto lobulado con aumento difuso de la matriz y lesiones nodulares mesangiales (flecha). A. Hematoxilina-eosina. B. Tricrómicro de Masson. 100x.

**Figura 2:**
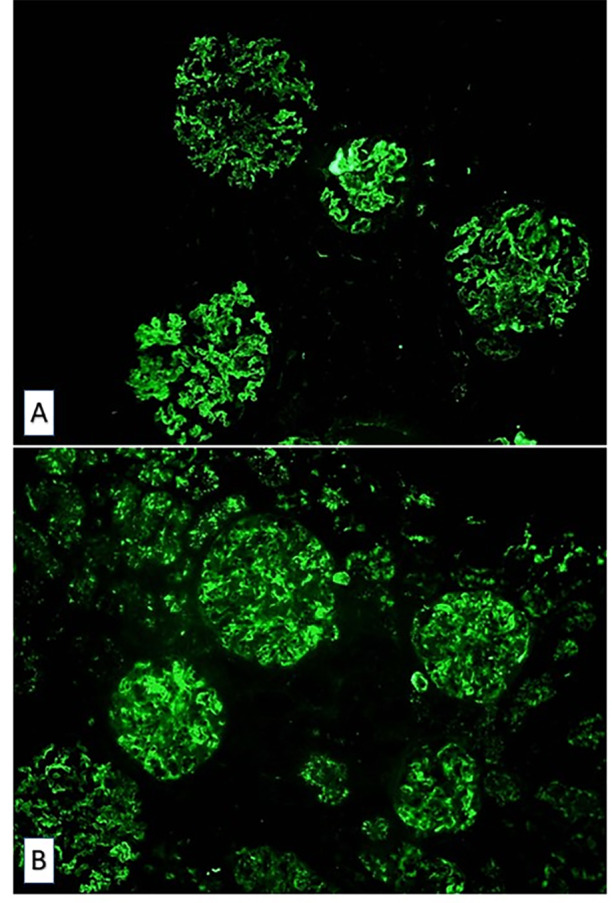
Microscopía de fluorescencia. A. Inmunofluorescencia para IgM. B. Inmunofluorescencia para C3. 100x.

En la biopsia renal se observaron glomérulos de aspecto lobulado con aumento difuso de la matriz, lesiones nodulares mesangiales (Fig. 1), con depósitos de inmunocomplejos y del complemento (Fig. 2), sin apreciarse semilunas ni hallazgos sugerentes de vasculitis. Estos hallazgos fueron compatibles con una glomerulonefritis membranoproliferativa mediada por inmunocomplejos secundaria a VHC. La biopsia cutánea mostró una dermatitis superficial perivascular. Se realizaron varias determinaciones de crioglobulinas durante el ingreso, siendo la tercera positiva.

Tras iniciar el tratamiento antiviral y coincidiendo con la disminución de la carga viral del VHC, la paciente experimentó una importante mejoría con remisión de la proteinuria, por lo que fue dada de alta y seguida en consultas externas de Nefrología.

Al cabo de ocho semanas, tras finalizar el tratamiento antiviral, la paciente presentaba una exploración física y analítica normal (Tabla 1), alcanzando una remisión completa del síndrome nefrótico; además, la carga viral de VHC se negativizó, por lo que fue dada de alta de consultas externas.

## DISCUSIÓN

Presentamos el caso de una mujer de 47 años con GnMP secundaria a una infección por VHC. La incidencia de casos similares ha disminuido notablemente en nuestro medio durante los últimos años^2^. El VHC es la causa más frecuente de GnMP (en torno al 70% de los casos)^1^^,^^5^^,^^6^.

La clínica en pacientes con GnMP es muy variada, desde una hematuria asintomática hasta un síndrome nefrótico con deterioro de la función renal, acompañado de hipertensión arterial y de lesiones cutáneas^1^, como fue en el caso de nuestra paciente.

Durante el ingreso en planta se solicitaron varias serologías (incluida la del VHC), ya que la GnMP puede estar causada por diferentes agentes infecciosos, incluyendo virus, bacterias u hongos^1^. También se realizó un estudio de autoinmunidad completo debido a que varias de estas patologías autoinmunes se relacionan con GnMP^1^. Además, se solicitó un estudio de electroforesis de proteínas tanto en sangre como en orina ya que, en los últimos años, varios estudios han vinculado la presencia de procesos monoclonales con el patrón histológico característico de GnMP^7^^,^^8^, por lo que es importante descartar que pueda tratarse del debut de un proceso linfoproliferativo oculto.

La GnMP se ha clasificada históricamente a partir del resultado del estudio de microscopía electrónica, dependiendo de la localización de los depósitos inmunes (tipo I: en mesangio y subendotelio, tipo II: en membrana basal del glomérulo y en túbulos, tipo III: subepiteliales, mesangiales y subendoteliales). Actualmente se realiza una biopsia renal^4^, en cuyo estudio microscópico se observó hipercelularidad mesangial, engrosamiento de la membrana basal glomerular e interposición mesangial en la pared capilar y glomérulos de aspecto lobulado, correspondiendo con la lesión histológica característica de este tipo de glomerulonefritis^1^. Posteriormente, se propuso una nueva clasificación en función de los hallazgos del estudio de microscopía de inmunofluorescencia, que permite observar el depósito de inmunoglobulinas y/o factores del complemento en la pared capilar y en el mesangio^1^, clasificando la GnMP en GnMP mediada por inmunocomplejos (depósito de inmunocomplejos y elementos del complemento) y en GnMP mediada por complemento (depósito de componentes del complemento en ausencia de inmunocomplejos)^4^. Nuestra paciente mostró depósitos de inmunocomplejos y, de forma similar a la mayoría de los casos secundarios a esta infección^5^, en la tercera determinación de crioglobulinas se obtuvo un resultado positivo.

El tratamiento de la causa de la GnMP es imprescindible para la resolución del cuadro.

La GnMP se asocia frecuentemente al VHC, siendo el tipo I, que se asocia con crioglobulinemia, el patrón histológico más frecuente. En estos casos el tratamiento va dirigido a eliminar la replicación viral, y a disminuir secundariamente la formación y el depósito glomerular de inmunocomplejos con el virus^5^. Para ello, se utilizan los antivirales de acción directa contra el VHC^5^^,^^6^, con buenos resultados en varios estudios que consiguieron, en la mayoría de los casos, una carga viral negativa entre las cuatro y seis semanas y la desaparición de las crioglobulinemias al cabo de cuatro meses^5^^,^^6^. En nuestro caso se pautó tres comprimidos al día de Glecaprevir/Pibrentasvir 100mg / 40mg porque puede administrarse en insuficiencia renal y su pauta es de ocho semanas en lugar de doce.

Los casos de menor gravedad, en los que no existe disminución del filtrado glomerular, se pueden tratar únicamente con antivirales^6^. Si la GnMP es debida a un proceso infeccioso, el uso de corticoides se reserva para cuando existe un deterioro grave de la función renal^2^^,^^6^. En este caso, y si el paciente es refractario al tratamiento con corticoides, se pueden utilizar otros inmunosupresores como la ciclofosfamida^2^^,^^3^. Algunos estudios recomiendan el uso de Rituximab, mejor tolerado que la ciclofosfamida, como tratamiento inmunosupresor de primera línea en los casos de GnMP secundaria a infección del VHC^6^^,^^9^, o en casos refractarios o que se acompañaban de un cuadro rápidamente progresivo^9^^,^^10^. En casos muy severos puede considerarse la plasmaféresis. Cuando se presenta un brote crioglobulinémico agudo, se recomienda el uso de Rituximab asociado a corticoides, administrado tras la plasmaféresis si es necesario el recambio plasmático^5^^,^^6^.

Debido a la gravedad de la situación inicial de la paciente (deterioro de la función renal y síndrome nefrótico con edema en EEII y proteinuria en rango nefrótico que llegó a superar los 8 g/L) y de acuerdo con otros estudios^3^, se inició tratamiento inmunosupresor con corticoides a dosis altas a la espera del resultado de la biopsia y de las serologías. La paciente mostró mejoría clínica y analítica, pero se valoró la administración de Rituximab en caso de mala evolución.

Al paciente que ha sido sometido a un trasplante renal, en caso de recidiva de GnMP secundaria a VHC se recomienda tratarlo de forma similar, con antivirales de acción directa e inmunosupresión si existe deterioro importante del filtrado glomerular^6^. En los pacientes con gammapatías monoclonales se debe iniciar el tratamiento propio de estas, y cuando exista una enfermedad autoinmune se recomienda inmunosupresión con Micofenolato y corticoides a dosis bajas para controlar la enfermedad^2^.

El pronóstico de los pacientes depende del grado de respuesta al tratamiento antiviral.

En conclusión, la GnMP es una nefropatía cada vez menos frecuente en nuestro medio, siendo la etiología más importante la infección por VHC. Para su tratamiento resulta fundamental conocer y resolver la causa subyacente que la está provocando mediante la administración de antivirales. Solo cuando existe un deterioro importante del estado general o del filtrado glomerular se recomienda el uso de corticoides y de inmunosupresores (Rituximab es el más recomendado).
